# Family forest owner’s perspectives on headwater streams in boreal forests: Motivations, values, and conservation actions

**DOI:** 10.1007/s13280-025-02163-3

**Published:** 2025-04-05

**Authors:** Mari J. Annala, Virpi Lehtoranta, Anu Hilli, Raili Hokajärvi, Minna Kuoppala, Heikki Mykrä, Sirpa Piirainen

**Affiliations:** 1 Finnish Environment Institute (Syke), University of Oulu, P.O. Box 413, 90014 Oulu, Finland; 2 Finnish Environment Institute (Syke), Latokartanonkaari 11, 00790 Helsinki, Finland; 3 Finnish Forest Centre, Aleksanterinkatu 18 A, 15140 Lahti, Finland; 4 Natural Resources Institute Finland (Luke), Yliopistokatu 6 B, 80100 Joensuu, Finland

**Keywords:** Biodiversity, Forest management, Forest guidance, Protection zone, Riparian, Water protection

## Abstract

Boreal headwater streams are vital for forest biodiversity and larger water bodies but face challenges from forest management. Understanding forest owner’s views is key for effective water management. Our research surveys family forest owners to explore: (1) their perception of stream value, (2) factors driving their willingness to protect streams, and (3) if motivations for forest ownership vary by residence. Most owners are motivated by recreation. Those living far from their forests prioritize landscape, heritage, and biodiversity more than nearby residents. While owners recognize the importance of biodiversity and water protection, this does not always influence their management choices. Owners who are familiar with the link between forest management and stream health are more willing to protect streams from logging impacts. These findings highlight the need for better advice and policies for forest owners on managing water quality and biodiversity.

**Supplementary Information:**

The online version contains supplementary material available at 10.1007/s13280-025-02163-3.

## Introduction

The Water Framework Directive obliges European Union (EU) states to take actions to achieve a good state of surface waters by 2027 (EUR-Lex 2000), and the Global Biodiversity Framework sets goals to improve the status of biodiversity (Convention on Biological Diversity 2024). However, the state of surface waters is largely poor in the boreal region (European Environment Agency 2021; Desforges et al. 2022) and EU member states have failed to achieve water management goals set by the EU (European Commission 2019). This failure also poses a challenge in meeting the recently set EU biodiversity targets (Biodiversity strategy for 2030; European Commission n.d.) and the Nature Restoration Law (EUR-Lex 2024), which states that 90% of degraded habitats, including surface waters, are to be restored by 2050. The failure to improve the state of surface waters is largely attributed to intensive land use (Härkönen et al. 2023; Räike et al. 2024). Headwater streams are sources of larger waterbodies and, as such, major contributors to water quality in rivers, lakes, and seas (Asmala and Scheinin 2024; Räike et al. 2024). Small headwater streams (with a catchment size under 15 km^2^) can make up 90% of the total river network length (Bishop et al. 2008), and thus have great potential regarding water quality and biodiversity in larger waterbodies. They play an essential role in maintaining the biodiversity of species specialized on shaded and well-oxygenized flowing waters (Richardson 2020). Streams also maintain the microclimate of their surrounding forests, making them distinctive habitats for riparian species and increasing regional forest biodiversity (Tolkkinen et al. 2020).

The ecological status of boreal streams has been largely impaired and is under further threat due to human activities (Janssen et al. 2016; WWF-Canada 2020). Forestry operations, such as soil preparation and peatland drainage, are among the main threats to headwater streams in forested lands (Sala et al. 2000). As headwater streams depend ecologically on the surrounding forests (Tolkkinen et al. 2020), harvesting of riparian zones poses a risk to aquatic ecosystems: currently, most riparian areas in the boreal zone are insufficiently protected (Kuglerová et al. 2020). In streams, the effects of past land use typically persist for decades, and despite active channel restoration measures and reforestation efforts, recovery is extremely slow (Harding et al. 1998), thus preventing deterioration in the first place is crucial if streams are to be safeguarded.

**Fig. 1:**
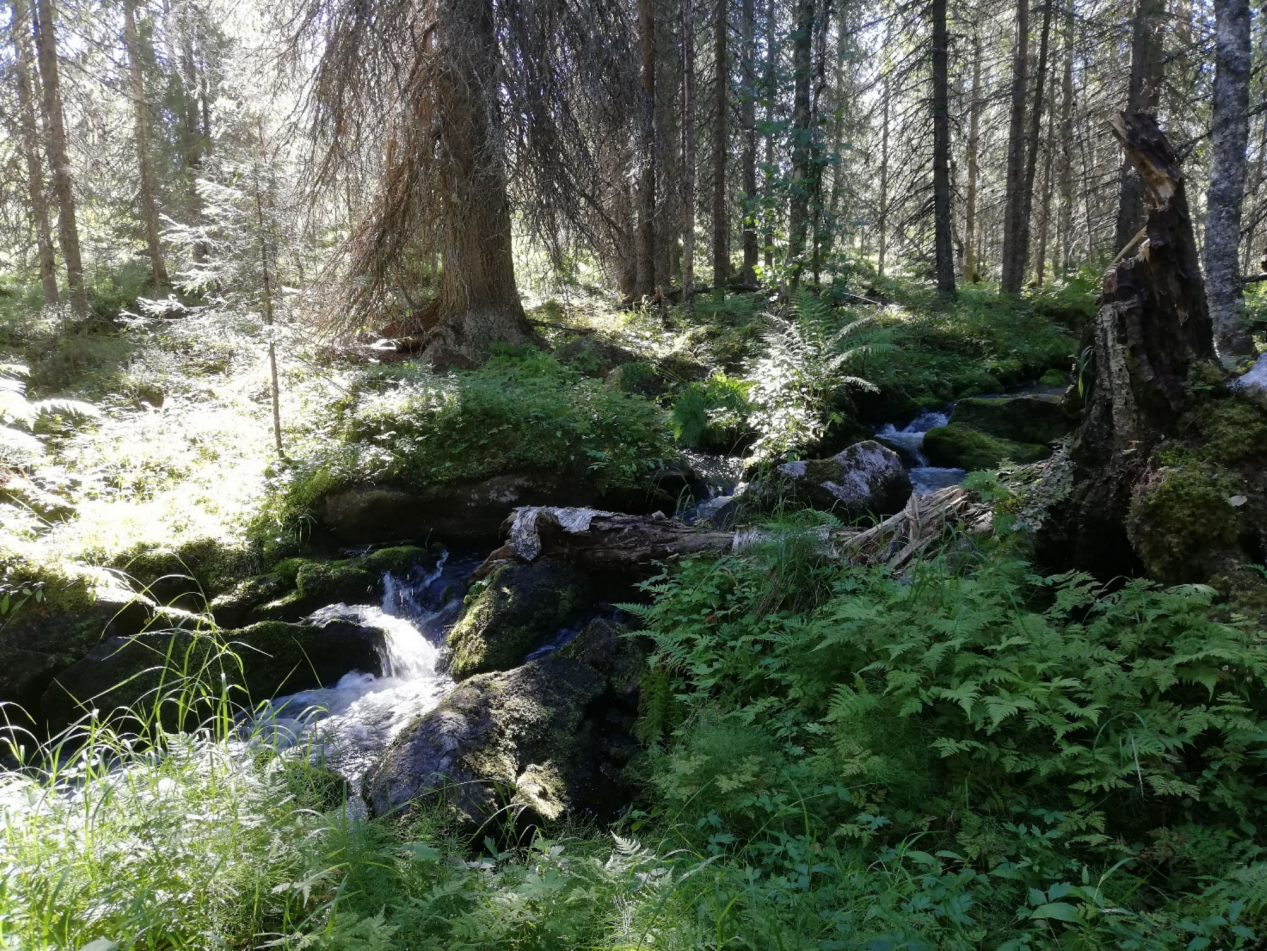
A near-natural stream in Northern Ostrobothnia. The forest in the catchment has been partly harvested, and there are some drainage ditches, but the stream channel and the riparian zone (buffer) are near pristine. The site is protected by Forest Act. Photo by M. Annala

Environmental awareness in forestry has gradually improved in Finland since the end of the 1960s-1970s (Siiskonen 2007). From a societal perspective, other forest-based ecosystem services, such as recreational use, have become important in addition to timber production (Winkel et al. 2022). At the same time, the demand for timber and woody biomass has increased due to their usage in bio-based industries and as a substitute for fossil fuels (Natural Resources Institute 2023). Given this development, forest management based on nature-based solutions in the riparian zones (e.g. Mykrä et al. 2023) has been incorporated into national recommendations for best practices in sustainable forest management (Tapio Oy n.d.) and to some degree, into forest certification systems (Programme for the Endorsement of Forest Certification (PEFC) 2024, Forest Stewardship Council (FSC) 2023). Nature-based solutions refer to maintaining, restoring, and rehabilitating natural processes and functions to enhance ecosystem services such as biomass production and water filtering (Keesstra et al. 2018). In contrast to these voluntary commitments, legal protection of headwaters is still weak. Only small-sized (< 2 ha, Finnish Forest Centre 2022) riparian areas with natural-like forest (Fig. 1) are protected against harvesting by the Forest Act (Forest Act 567/2014), and the Water Act (Water Act 587/2011) still enables ditch network maintenance measures that compromise water protection targets (Härkönen et al. 2023). Compared to other Nordic (Norway, Denmark, Iceland) and Baltic (Estonia, Latvia) countries, where stream protection is well regulated by legislation, Finland and Sweden (along with Canada) rely largely on voluntary commitments on riparian buffer retention (Ring 2017; Kuglerová et al. 2020). Thus, especially in the latter countries, certification systems have a great potential to play a critical role in determining how sustainable forestry practices are (Hysing and Olsson 2005). In Finland, about 94% of managed forests (excluding poorly productive and unproductive land) are certified in the PEFC system (PEFC 2023). According to the PEFC Finland standard, a riparian buffer that is on average at least 10 m wide and the minimum width of which is everywhere at least 5 m is left along natural and near-natural state streams. PEFC allows selection felling on the buffer zone. Compared to PEFC, the more ambitious certificate in terms of ecological sustainability, the FSC Finland system, covers about 9% of the forests and requires a 30 m unharvested buffer zone along streams that are in natural or near-natural state (Natural Resources Institute 2020; FSC 2023). Danley et al. (2021) point out that certification schemes and other voluntary-based methods have only limited ability to enhance biodiversity and water protection, which raises an interesting question of whether certification can prevent the generalization of ecologically sustainable riparian buffers.

The Nordic countries have a high proportion of privately owned land, ranging from 70 to 86%, compared to 6% in Canada (Ring et al. 2017; Stinson et al. 2019). In Finland and Sweden, 52% and 49%, respectively, of the forestry land is privately owned by individuals, families, private partnerships, or estates of deceased persons (Siiskonen 2013; Skogsstyrelsen 2023; Vaahtera et al. 2023). Therefore, the attitude of these private owners, later called as family forest owners, towards recommendations and instructions plays a crucial role in achieving the target of improving the status of surface waters as required by the Water Framework Directive (EUR-Lex 2000) as well as goals to improve the status of biodiversity required by the Global Biodiversity Framework (Convention on Biological Diversity 2024). Their interest towards water and biodiversity protection, as well as their preferences for forest management practices, is essential for voluntary nature-based solutions to become more common practices and for the acceptance of common policies, regulations, and standards set by authorities.

**Fig. 2:**
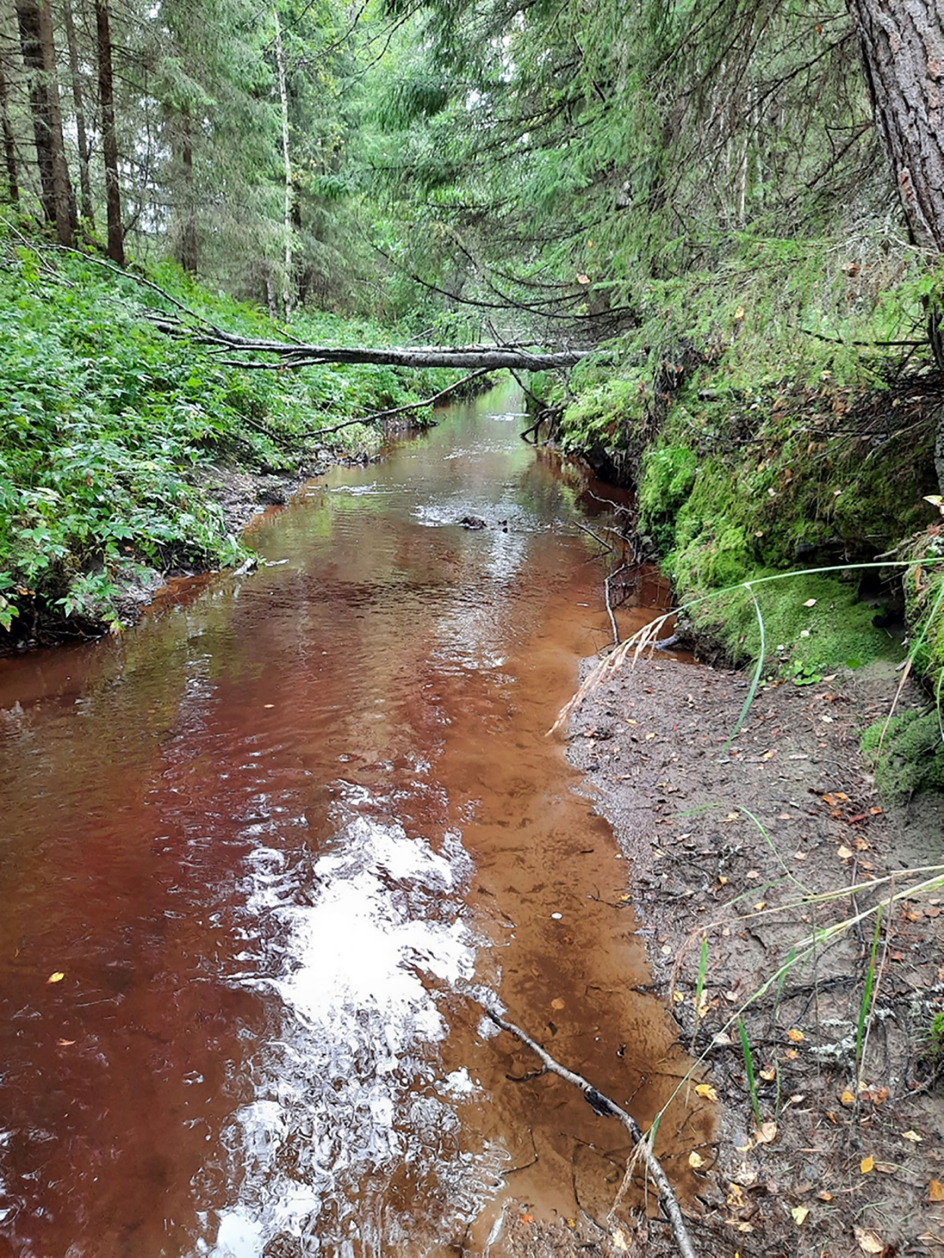
A degraded stream in Northern Ostrobothnia. The stream has been channelized and cleaned of boulders, and the catchment has been drainage ditched. The forest in the riparian zone has changed due to past harvestings. Photo by M. Annala

According to a recent modelling of physical condition of stream channels, there is over 100 000 km of streams in Finland with a catchment size under 100 km^2^, of which about 85% have been degraded primarily due to forestry practices (Finnish Environment Institute 2021; Appendix S1; Fig. 2). However, we do not know how streams are valued by forest owners, who are largely deciding on the future of these unique habitats.

Several surveys about Finnish family forest owners´ objectives have been published since the end of 1990s, focusing on their general aims and forest management preferences. Those studies indicate that while the forest owners of twentieth century were multi-objective, the proportion of owners who stress non-economic forest values has increased since then (Kangas and Niemeläinen 1996; Kendra and Hull 2005; Toivonen et al. 2005; Tikkanen et al. 2006; Leppänen 2010; Häyrinen et al. 2015; Häyrinen 2017; Muttilainen et al. 2023). Economic owners refer to those forest owners whose principal objective in forest ownership is obtaining income through wood trade; multi-objective owners resemble those who have several aims regarding the forest, such as recreational use, biodiversity protection, and economic use. In contrast, Bergseng and Vatn (2009) show that Nordic forest owners emphasize direct forest use values (i.e. economically oriented owners) although they are positive towards forest biodiversity protection in general (i.e. positive attitude towards biodiversity protection yet not necessarily on one´s own land or without economic compensation). The educational level, source of income, and age of forest owners have been identified as explanatory factors for their aims (Tiebel et al. 2022). Specifically, more educated, younger, and non-farmers tend to be more supportive of less intensive forest management practices than less educated, older, and farmers (Hallikainen et al. 2010; Juutinen et al. 2020; Husa and Kosenius 2021). However, owners over the age of 65 may cut less forest than younger possibly due to heritage motives (Kuuluvainen et al. 1996; Kuuluvainen and Tahvonen 1999; Koskela and Karppinen 2024).

The effect of place of residence on forest management decisions is controversial: Typically, those living further from there forest estate, especially citizen forest owners, have been noted to emphasize recreational values, biodiversity and/or conservation more than rural owners (Häyrinen et al. 2015; Tyrväinen et al. 2021), although this is not always the case (Uliczka et al. 2004; Favada et al. 2009; Nordlund and Westin 2011). Distance to forest estate may also explain how forest owners´ make decisions regarding their forest holdings; local owners being more often independent managers compared to distant owners (Hujala et al. 2009; Karppinen and Berghäll 2015; Juutinen et al. 2020). As the proportion of urban owners is increasing (Leppänen 2010; Karppinen 2012; Živojinović et al. 2015), the effect of place of residence on attitudes towards stream protection should be studied in more detail to enable targeted guidance or training to forest owners effectively. Indeed, former studies indicate that there is a communication gap among forest owners, forest professionals, authorities, and researchers (Bergseng and Vatn 2009; Korhonen et al. 2013; Häyrinen et al. 2015; Vainio and Paloniemi 2012; Kuhlman et al. 2022; Takala et al. 2022), which may complicate cooperation.

Studies exploring attitudes towards protection of riparian habitats have mainly focussed on the willingness of the general public to pay for maintaining ecosystem services or enhancing habitat protection or restoration. Of these, relatively few specifically consider water protection or water-related ecosystem service targets (Kreye et al. 2014; Lehtoranta et al. 2017; see also Lewis et al. 2017; and Aguilar et al. 2018 for non-boreal studies). Research considering the family forest owners attitude towards stream or riparian protection is scarce and mainly conducted in a non-boreal zone in United States (Kline et al. 2000; Dutcher et al. 2004; Janota and Broussard 2008; Cooper and Jacobson 2009; Kang et al. 2019). However, Mancheva (2018) surveyed the willingness of individual forest owners to participate in collaborative river basin governance in the Krycklan catchment, Sweden. The results were unambiguous as over half of the landowners were unwilling to collaborate. Mancheva (2018) stated that the low interest stemmed from the fact that respondents did not consider water quality as important, they lacked knowledge on how waters should be considered in forestry operations, they did not see the value of their own property and actions for water protection, they had low trust in public authorities and private forest companies compared to other forest owners, and there was no one taking the leadership in enhancing water protection in the catchment. Also, financial compensation may increase the desire to use less-intensive management measures (Matta et al. 2009; Boon et al. 2010), and the knowledge base of forest owners may affect their interest towards the state of biodiversity and enhancing water quality (Lähdesmäki and Matilainen 2014; Eriksson and Fries 2020). Thus, it is important to consider ways to reach and involve the forest owners (Korhonen et al. 2013; Matilainen et al. 2023). Furthermore, forest certification plays a role in determining the standards for riparian buffers. Overall, so far, the certificates have been insufficient to make up for the shortcomings of legal requirements in water protection (Villalobos et al. 2018; European Commission 2019; Jyväsjärvi et al. 2020; European Environment Agency 2021; Desforges et al. 2022).

Here, we study the views of family forest owners about headwater streams through a survey and quantitative analyses. Our research questions are: (1) how do forest owners value streams and are they aware of the actual state of the streams, (2) what explains the willingness to take stream protection actions that exceed forest management guidelines, national restrictions, and forest certification criteria, and (3) does the motivation of forest ownership differ across place of residence.

## Materials and methods

The survey was conducted in North Ostrobothnia, which is situated in northern Finland (66.4858–63.4291°N, 22.7789–30.1384°E) and covers 39 193 km^2^ of land area (11,6% of Finland), with 32 550 km^2^ being forestry land (Niinistö et al. 2023). This region was chosen as the study region as it represents the average of the whole country in many ways: for example, the state of streams, the average area of forest estates owned by families, and the average age of owners in the region is close to the national average (Finnish Environment Institute 2021; Finnish Forest Centre 2024).

The electronic questionnaire (Appendix S2) was sent in April 2022 to forest owners (n = 14 759) who owned more than 10 hectares of forest in the study area either by themselves or with their spouse, via private partnerships, or jointly by their estates. The e-mail addresses were obtained from the Finnish Forest Centre’s register of forest owners. These corresponds to about 60% of family forest owners in North Ostrobothnia. The questionnaire with 22 questions was first tested by sending it to a sample of 203 forest owners in the area. After testing, small changes were made, such as defining the terms ‘buffer zone’ and ‘riparian forest’. After 11 days of initial sending, the questionnaire was sent again to those respondents who had not yet answered.

We incorporated several questions related to the background of the respondent and her/his forest property. The questions on the personal background included information such as age, education level, childhood residence place (urban/rural), gender, and involvement in forestry or agriculture. The questions on details of the forest property concerned, e.g. the area and the protection percentage of the holding(s) and whether the property has been certified or not. We used these factors to study the special characteristics of forest owners on the study region and to validate how well our sample resembles the population of forest owners in North Ostrobothnia. Further, we used these factors to analyse which of them, if any, are related to decisions made by the respondent regarding riparian forest management.

To allow focusing some of the analyses on riparian forest owners, we asked if any of the holdings owned by the respondent bordered surface waters and which kind. To avoid potential variable interpretation, we defined that a ‘forest holding bordering to surface waters’ means that the holding is located 30 m at maximum from the waterbody. Streams were defined as having an average channel width 5 m or less to distinguish from rivers.

As the motivation to own forest can greatly influence how biodiversity promotion and water protection are perceived, we included a question on how much forest-related subjects influence the desire to own a forest. We requested the respondent to provide an answer on a five-point scale ranging from ‘not at all (1)’ to ‘very much (5)’ in response to 13 given statements. One of our main objectives was to understand the opinions and perceptions of forest owners regarding forest streams. A set of statements were presented, related to the values of landscape, biodiversity, quality, and meaning to human well-being of the streams, how these are affected by forestry activities, and what is the responsibility of the forest owner in relation to stream state and biodiversity. The respondent was asked to indicate agreement or disagreement with each statement on a seven-level scale from ‘strongly disagree’ to ‘strongly agree’, with an option of ‘don´t know’. We also included a specific statement about the state of the streams to assess if the forest owners are aware of the actual overall state of the streams in the area.

Furthermore, we were interested in what explains the willingness to take stream and riparian protection actions beyond common guidelines, national restrictions, and forest certification criteria. For this, we included several questions about the future of riparian management on the questionnaire. The width of the buffer zone in future loggings along streams was elicited with a question: ‘If there is logging planned along the stream in your area, what kind of protective buffer zone would you prefer? Please select the most likely option’: ‘No logging planned along the stream’, ‘Variable width, averaging 10 m, minimum 5 m’, to ‘Uniform 30 m’, and ‘Other, please specify’.

For the following analyses, we considered a sub-sample of respondents who owned forest along a stream (n = 1322). An ordered probit model was used to analyse which background variables were associated with wider buffer zones. In the model, respondents’ choice (dependent variable) was coded using a 3-point response scale from a buffer zone width of 0-5 m (1), at least 5 m (2), or at least 15 m (3). From the dataset under analysis, 400 respondents who indicated that there would be no logging along the stream in their forest holding were removed. We then conducted regression models to find out which explanatory factors affect forest owners´ choice of wider buffer zones and management intensity of the buffer zone in future loggings.

Next, respondents were asked how they would prefer the forest in the buffer zone to be managed if there was logging planned along their stream. The respondents could choose multiple options, which included: ‘No logging planned along the stream’, ‘All trees are left in the buffer zone’, ‘Thinning of the tree stand can be done in the buffer zone’, ‘Selective cuttings can be done aiming for natural regeneration of the tree stand in the buffer’, ‘Retention tree groups are concentrated in the buffer zone’, ‘All deciduous trees, understory, and shrub layer are saved in the buffer zone’, ‘Only single retention trees are left in the buffer zone’, ‘The proportion of deciduous trees is increased in the stand of the buffer zone’, and ‘Other, please specify’. A probit model with an ordinal dependent variable was used to assess the intensity of loggings in the buffer zone, where the quantity of trees ranged from ‘Remove all trees in the buffer zone’ or ‘Concentrate retention tree groups in the buffer zone’ (1) to ‘All trees are left in the buffer zone’ (4). Responses with ‘No logging planned along the stream’ and empty responses were removed from the analysis (n = 353 and n = 223, respectively).

We performed t-tests to compare differences in objectives of forest ownership between forest owners living outside the municipality of their holding. An ordered probit models were employed to estimate the factors influencing the probability of choosing wider buffer zone and lower logging intensity in future. The statistical software SPSS (27) was utilized for all statistical analyses.

## Results

### Response rate and the characteristics of forest owners in the study region

Two rounds of online surveys resulted in a total of 2140 responses, achieving a response rate of 14.5%. This compares well with the latest response rates of online surveys to family forest owners (Karppinen et al. 2020; Koskela and Karppinen 2021). The statistics of age, gender, size of owned forest area, and proportion of estates owned by heirs among the respondents were well aligned with those of the population (family forest owners in North Ostrobothnia), excepts for gender, which was skewed towards male respondents (21% of women respondents vs. 25% of women in the population). This indicates that despite the relatively low response rate, the data adequately represents the opinions of the population (for further discussion of data representativeness, see Discussion).

Half of the respondents (50%) lived outside of the municipality where they owned forest. The others either resided in the same municipality as their holding (34%) or on their holding (15%). Most respondents (69%) had recently made timber sales, and more than one-fourth of respondents (28%) owned protected forest land. Half of the respondents (52%) stated to have streams and 30% stated to have rivers in their forest. The descriptive statistics of the responses are presented in Table 1.

**Table 1:** Descriptive statistics for all respondents, the sub-sample of respondents used in the regression models, and all family forest owners in the area (mean = arithmetic mean, SD = standard deviation). Abbreviation n.a. stands for “data not available”

Factor	All respondents		Respondents owning forests along streams and rivers		Forest owners of North Osthrobothnia
	Mean	SD	Mean	SD	Mean
Average age	59 y	12.2	59 y	12.2	60.5 y
Gender; proportion of women respondents	21%	0.4	19%	0.4	25%
An 'urban childhood landscape'*	22%	0.4	22%	0.4	n.a
Academic education	21%	0.4	23%	0.4	n.a
Farmer or forestry entrepreneur	45%	0.5	47%	0.5	n.a
Living permanently on the holding	15%	0.4	16%	0.4	n.a
Living outside the municipality of the holding	50%	0.5	50%	0.5	n.a
Size of owned forest area	94 ha	152.7	115 ha	185	100.6 ha
Family owner—by her/him self	52%	0.5	49%	0.5	n.a
Family owner—with one or several persons	25%	0.4	26%	0.4	n.a
Family owner—private partnership	17%	0.4	20%	0.4	n.a
Family owner—estate owned jointly by heirs	3%	0.2	4%	0.2	4%
Made timber sales over the last 3 years	69%	0.5	71%	0.5	n.a
Size of conserved forest area in the holding	3.0 ha	15	4.1 ha	18.5	n.a
Stated to have streams in the forest	52%	0.5	85%	0.4	n.a
Stated to have rivers in the forest	30%	0.5	51%	0.5	n.a
Does not know if forest has been certificated**	26%	0.4	25%	0.4	n.a
‘My forest has been certificated by FSC’	7%	0.3	7%	0.3	n.a

^*^ In the questionnaire the main place of residence during childhood was asked

^**^ Either PEFC or FSC or both

### Forest ownership objectives

Recreational use of forests was identified as the primary motivator for forest ownership, with respondents who permanently lived on their holdings attributing this factor as the most important selection criterion the most often (47% of respondents; Table 2). The objectives of forest ownership differed to some extent between those living outside the municipality of their holding and those who lived on their holdings or in the same municipality as their holdings. For family forest owners who lived outside the municipality of their holding factors such as ‘landscape values’, ‘legacy for children’, ‘conservation of biodiversity’, and ‘forest as an investment’ were more common motivators than benefits related to everyday use of the forest, such as ‘firewood supply’ and ‘exercise in working in the forest’. Economic and recreational value of the forest was a more significant factor in motivating owners who lived on their holdings compared to those living in the same municipality as their holdings or further (*t* = 4.764, *p* < 0.001, and *t* = 2.563, *p* = 0.011, respectively).

**Table 2:** The seven most important values in respondents’ desire to own forest, across place of residence. Recreational use includes berry picking, hunting, nature walks and outdoor creation. Conservation of biodiversity means e.g. leaving decaying trees in the forest

Permanently on the holding n = 313–317	Elsewhere in the same municipality as the holding n = 724–733	Outside the municipality where the holding is located n = 1053–1070
1. Recreational use of forests, 47%	1. Recreational use of forests, 40%	1. Recreational use of forests, 38%
2. Firewood supply, 46%	2. Firewood supply, 34%	2. Legacy for children, 27%
3. Benefits of exercise in working in the forest, 37%	3. Benefits of exercise in working in the forest, 30%	3. Landscape, 25%
4. Landscape, 34%	4. Landscape, 24%	4. Benefits of exercise in working in the forest, 23%
5. Financial security in case of unexpected expenses, 27%	5. Legacy for children, 22%	5.-6. Firewood supply / Conservation of biodiversity, 19%
6. Legacy for children, 26%	6.-7. Financial security in case of unexpected expenses/ Conservation of biodiversity, 18%	7. Investment, 18%
7. Main source of income or regular additional income, 25%		

### The attitudes of forest owners towards streams and their protection

**Fig. 3:**
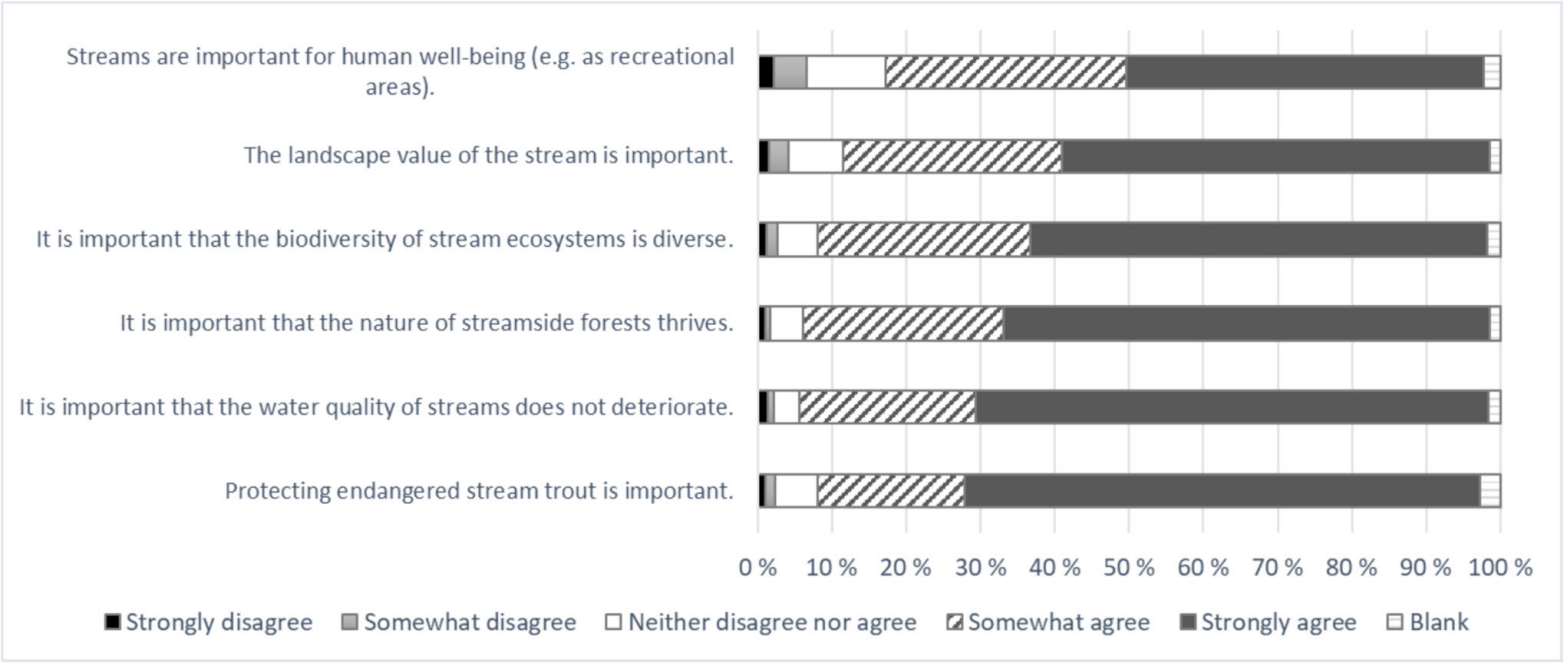
Attitudes towards values related to forest streams (n = 2140)

Over 90% of family forest owners acknowledged that stream biodiversity, water quality, and the good condition of riparian forests are important (Fig. 3). Protecting endangered trout in the study area was also considered important by almost 90% of the respondents. The landscape and recreational values of streams were also deemed important by a large proportion (> 80%) of forest owners.

**Fig. 4:**
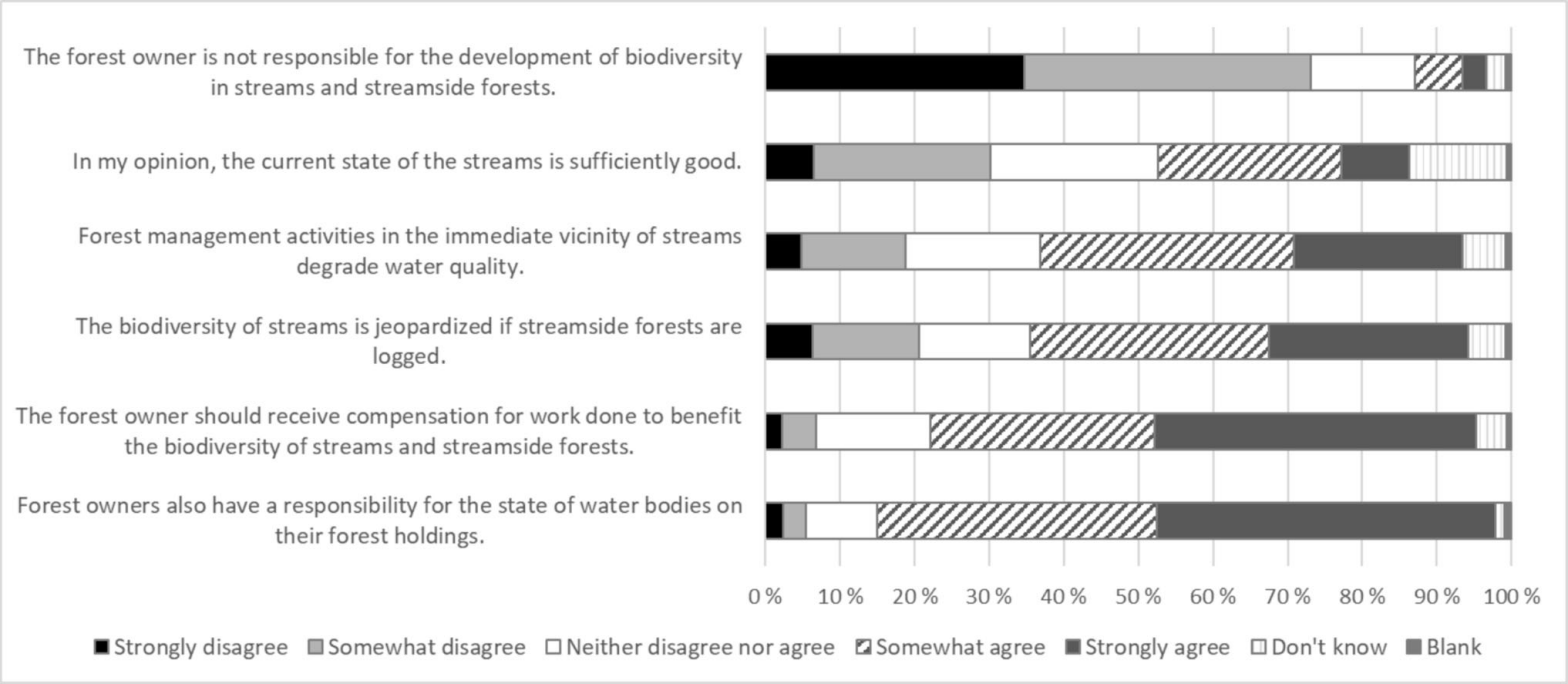
Perceptions of the impacts of forestry activities on stream ecosystems (n = 2140)

Most forest owners acknowledged their role in enhancing biodiversity in streams and riparian forests (73% agreed to some extent; Fig. 4). Also, a significant majority of respondents (82%) believed that it is the responsibility of the forest owner to contribute to maintaining good stream status on their forest holdings, but they acknowledged that incentives would be needed (73% agree to some extent). However, the level of knowledge about stream state and how forest management affects streams was lower. The first mentioned caused divided opinions; almost one-third (30%) of forest owners thought that the state of streams in North Ostrobothnia was good enough, while a similar proportion disagreed (30%). A little less than 60% understood that forest management actions near the streams can jeopardize stream biodiversity and water quality, while about 20% held the opposite view.

### Factors explaining future streamside loggings

Over half of the respondents (62%) reported owning forest along streams or rivers (Table 1). After testing the significance of various socio-economic and attitudinal factors, the final model for predicting the choice of a wider buffer zone included four factors, all of which were statistically significant at *p* < 0.10 (Table 3). The most significant factor affecting respondents likelihood to choose a wider buffer zone for future streamside logging was their opinion that streamside logging impacts stream quality and biodiversity (*IMPACTS* in Table 3). The desire to obtain information on alternative forest management practices and recommendations for enhancing forest biodiversity was also a significant factor (*INFORMATION* in Table 3). Wider buffer zones were also more likely to be chosen by respondents who had left at least 15 m of equal-width buffers along streams in previous loggings (*BUFFER* in Table 3). Finally, living outside the holding municipality was associated with choosing wider buffer zones for future streamside logging (*RESIDENCE* in Table 3). The Nagelkerke R^2^ measure of 0.321 indicates good explanatory power of the model.

**Table 3:** Ordered probit model with statistically significant explanatory variables on the width (1–3) of buffer zone in future loggings along streams. χ^2^ (df = 4) = 158.03 (p < 0.001), -2 Log Likelihood = 197.05 (p < 0.001), N = 607

Variable	Variable description	Coefficient (Standard error)	Wald statistics
*RESIDENCE*	Living outside of the municipality of the holding (yes = 1, no = 0)	0.24 (0.12)*	4.18
*BUFFER*	Has left at least a 15 m equal width buffer along streams during earlier loggings holding (yes = 1, no = 0)	0.76 (0.21)***	13.33
*IMPACTS*	Degree of accepting the statement ‘Water quality is degraded by forestry operations along the stream’: Totally disagree (1),…Totally agree (5)	0.51 (0.06)***	72.18
*INFORMATION*	Degree of accepting the statement ‘I actively seek information about available knowledge and/or the latest recommendations related to promoting forest biodiversity.’: Totally disagree (1),…Totally agree (5)	0.21 (0.074)***	7.99

^***^p < 0.001, **p < 0.01, *p < 0.1

The factors used in the final model for choosing lower logging intensity included six factors, all of which were statistically significant at *p* < 0.10 (Table 4). The most significant factor affecting the respondents´ likelihood of leaving more trees in the buffer zone in future logging was their level of agreement with the statement ‘Water quality is degraded by forestry operations along the stream’ (*IMPACT* in Table 4). Older age (*AGE*), not having loggings along the stream during their ownership (*LOGGING*) and agreement with the statement ‘Nature conservation influences my desire to own a forest’ (*NATURE*) were also factors associated with leaving more trees. Interestingly, being unaware whether their forest was certified by either the PEFC or FSC certification scheme increased the likelihood of leaving more trees in the buffer zones in future loggings (*CERTI*). As expected, if the respondents stated that they had habitats protected by the Forest Act along a stream (*FORESTACT*), they were more likely to leave more trees in the stream buffer zones in future logging operations compared to respondents not owning Forest Act habitats.

**Table 4:** Ordered probit model with statistically significant explanatory variables on the management intensity (1–4) of the buffer zone in future loggings along streams. 1 = high logging intensity (full cut to shrubs and retentions trees left on the buffer), 2 = multi-objective favouring broad leaved trees, 3 = multi-objective favouring continuous cover forestry or similar management, 4 = no logging on the buffer. χ^2^ (df = 6) = 135.98 (p < 0.001), -2 Log Likelihood = 1439.87 (*p* < 0.001), *N* = 697

Variable	Variable description	Coefficient (Standard error)	Wald statistics
*IMPACT*	Degree of accepting the statement ‘Water quality is degraded by forestry operations along the stream’: Totally disagree (1),…Totally agree (5)	0.29 (0.042)***	48.25
*NATURE*	Degree of accepting the statement ‘Nature conservation influences my desire to own a forest’: Not at all (1), Very much (5)	0.14 (0.043)**	10.21
*CERTI*	Stated not to know whether his/her forest is certified under the PEFC or FSC certification system (1); Other (0)	0.19 (0.11)*	3.35
*FORESTACT*	Stated to have Forest Act sites along a stream (1); Other (0)	0.14 (0.087)*	2.75
*LOGGING*	Loggings has been done along streamside during respondent´s ownership (1); Other (0)	− 0.14 (0.067)*	4.4
*AGE*	Age of the respondent	0.01 (0.004)**	7.41

^***^p < 0.001,**p < 0.01,*p < 0.1

## Discussion

Family forest owners possess 52% of Finnish forestry land and thus their decisions have a major impact on the state of headwater streams. Through a multi-choice questionnaire approach, we found that forest owners highly value nature and consider biodiversity and water protection of streams to be important. However, this was not always directly reflected into their forest management choices. The perception of how forest management impacts stream water quality was the most influential factor in explaining forest owners´ willingness to increase riparian buffer widths and to adopt less intensive logging practices in the future. This highlights the need for improved guidance on the environmental impacts of forest management, particularly regarding water quality, to improve the status of streams and riparian forests.

Recreational use of forests was the most common motivator for owning a forest, which may align with biodiversity protection values. For instance, enhancing stream protection by implementing wider and more wooded buffers, such as using continuous cover forestry, can benefit recreational use of stream areas due to, e.g. increased habitat quality for fish and improved aesthetic values. In Florida, USA, recreationally oriented forest owners were more willing to adopt a 60-m riparian buffer zone with less financial compensation than owners emphasizing forest investment, timber production, wildlife, or those with undefined objectives (Kline et al. 2000). However, recreational use of forest can be contradictory to biodiversity and water protection if it leads to actions such as maintaining open riparian zones and clearing stream of fallen trees (Tolkkinen et al. 2020).

In addition to their recreational value, forests are perceived as a source for economic security of owners or their children and are valued for their contribution to the landscape. This somewhat aligns with former studies that have shown 21st-century family forest owners to be multi-objective (Kline et al. 2000; Kendra and Hull 2005; Toivonen et al. 2005; Tikkanen et al. 2006; Leppänen 2010; Nordlund and Westin 2011; Häyrinen et al. 2015; Häyrinen 2017; Muttilainen et al. 2023). This is not a novel finding: since almost three decades ago, Kangas and Niemeläinen (1996) concluded that economic gains were not the primary values for most Finnish forest owners (excluding large forest estate owners) or the general public. The proportion of economically oriented owners has decreased further since then (Leppänen 2010). However, focusing primarily on economic values cannot be directly linked to the level of forest management intensity. In fact, several studies indicate that forest owners with multiple objectives are the most proactive in managing their forests for timber or biomass production (Kuuluvainen et al. 1996; Karppinen 1998), yet contradictory results have also been reported (Hallikainen et al. 2010).

Forest owners’ place of residence affected their attitude towards biodiversity and somewhat affected their motivation for owning a forest. Recreational use as a motivator for forest ownership seemed to be universal regardless of place of residence. However, owners residing outside the municipality of their forest holding emphasized the importance of landscape, heritage to children, and biodiversity conservation more than those living proximity to or on the holding, as was observed by Häyrinen et al. 2015, and Tyrväinen et al. 2021 (but for contrasting results, see Uliczka et al. 2004; Favada et al. 2009; Nordlund and Westin 2011). Nonetheless, we cannot conclude that biodiversity is irrelevant to owners living in proximity to or on their forest holdings; other factors associated with daily life may simply override biodiversity considerations. This is supported by the fact that these owners emphasized firewood supply and benefits of exercise in working in the forest.

Family forest owners in North Ostrobothnia share a common desire to maintain the good condition and high biodiversity values of forest streams. Similar responsibilities were detected among United States riparian forest owners (Janota and Broussard 2008). However, it is important to note that the concepts of “biodiversity” and “stream state” may mean different things to people. Also, there may be a tendency to agree with our propositions as they are presented in a way that does not require action. However, there appears to be more ambiguity and varying attitudes regarding the effects of forest management methods on state and biodiversity of streams. It is widely acknowledged that clear-cutting and soil preparation near a stream have a significant negative impact on the stream ecosystem (Tolkkinen et al. 2020), so there appears to be a lack of transfer of research results to silvicultural practices. Further, we inquired as to whether the overall stream state is good enough in the study area, leading to strongly polarized responses. The concept of “good enough” is vague but compared to the actual state of streams in the region, the knowledge gap regarding the identification of characteristics that define a natural stream becomes apparent. In the study region, only one third of headwater stream sections are in a natural or near-natural state (Finnish Environment Institute 2021). The degradation of a stream, and indeed the overall state of streams, may not be easily interpretable to non-expert forest owners. This is supported by the fact that a significant proportion of the respondents did not know (25%) or were not willing to express their opinion on the question about stream state (22%). However, the state of streams seems to be better understood or accepted than the need to consider biodiversity seriously as only 21% of forest owners worried about biodiversity loss in forests in the study of Takala et al. 2022. In contrast, according to a survey conducted by Koskela and Karppinen (2024), half of forest owners were strongly committed to protecting biodiversity in their forests in the near future (and one-fourth had weak intentions).

Our results suggest that forest owners have a variety of factors influencing their decisions about riparian forest management. Firstly, forest owners residing further from their forest holdings were more inclined to leave wider buffers than local owners. This may be attributed to the fact that far residing owners, compared to local ones, were more motivated by the aesthetic (landscape) and biodiversity values of forest ownership–yet landscape was also rated among the top four reasons for local owners to own forest. Hence, the appreciation of biodiversity values may explain the preference for wider buffers beyond the other mentioned factors. This is supported by the fact that forest owners were more willing to leave wider buffers when they perceived nature protection and water quality as important and understood buffer zones as crucial tools for preserving nature values. Additionally, respondents who portrayed themselves as active seekers and users of information were keener on employing wider buffers than those who were passive (see also Lähdesmäki and Matilainen 2014 and Eriksson and Fries 2020).

In addition to considering the width of the buffer zone, it is important that trees and shrubs, providing shading and a food source for stream and riparian organisms, are left uncut. The willingness of forest owners to retain trees on a buffer zone was higher among those who embraced conservation and understood the risks of conducting forest management practices near streams. This highlights the need for efforts to inform and train forest owners. Moreover, our results indicate that forest certification systems play a part in shaping forest owners´ opinions and their management approaches. Those respondents who did not know whether their forest was certified or not were more willing to retain trees than the owners who knew their forest was certified. According to Danley (2018), the readiness of owners of non-certified forests to set aside forest from harvesting is higher among those who believe that collective action is needed to safeguard biodiversity compared to those who think it is the state´s responsible to reach conservation goals. The fact that almost all our respondents agreed that the forest owner is partly responsible for biodiversity may explain our result. On the other hand, forest owners may be inclined to believe that the certification scheme will provide adequate protection, which is not necessarily the case (Villalobos et al. 2018; Jyväsjärvi et al. 2020), and they thus may misjudge the ecological sustainability promises of the certificate. Thus, the importance of incorporating water and biodiversity protection into forest guidance becomes even more crucial. Owners who had retained trees in previous logging operations were planning to do so in the future as well. This holds promise that once the forest owners are informed and convinced of the benefits of closer-to-nature forest management decisions, their attitude tends to remain permanent.

Our results suggest that older forest owners are more willing to employ less intensive forest management practices on buffers compared to younger ones. This is somewhat surprising and seemingly contradictory to the results of Juutinen et al. (2020), who found that older forest owners tend to allocate more land to traditional even-aged forestry rather than continuous cover forestry. Our result also contrasts with the findings of Husa and Kosenius (2021), who detected that younger and more educated owners were more supportive of less intensive management practices. On the other hand, our results align with earlier studies by Kuuluvainen et al. (1996) and Kuuluvainen and Tahvonen (1999), which are supported by a recent literature review by Karppinen (2012). Kuuluvainen et al. (1996) point out that the motivation behind this phenomenon could be legacy as aged owners may wish to leave their forest as an inheritance. Additionally, younger owners may require forest income more than their older counterparts, e.g. if they are starting their careers and potentially starting families. Similar results were obtained in a recent study, which suggests that active timber production, compensation levels, small forest ownership, and the willingness to leave forests as inheritances can hinder forest owners´ intentions to safeguard biodiversity more than do their attitudes towards biodiversity or stakeholder pressure (Koskela and Karppinen 2024).

Our results indicate that family forest owners value forests especially for their recreational and aesthetic qualities. They are willing to protect streams and riparian forests if they are provided with information about the benefits of employing closer-to-nature forest management practices and are given the freedom to make decisions regarding their property. However, the common sentiment was that protection efforts should be financially compensated for. This is not surprising as offering compensation often enhances forest owners´ willingness to set aside forest for conservation (Matta et al. 2009; Boon et al. 2010). Notably, they require a more comprehensive forest advisory service that provides information on the effects of various forest management practices on biodiversity and water protection. Providing training and access to relevant information to forest owners is crucial. It is also important to train forest advisors and specialists, who are the most efficient distributors of information among forest owners (see also Korhonen et al. 2013). Expanding the range of means to manage forests could strengthen the credibility of the advisors and create trust between forest owners and advisors. As they operate close to forest owners, information of safeguarding nature is probably more efficiently distributed to most forest owners from forest specialists such as Forest Management Associations and timber buying companies (Hysing and Olsson 2005; Korhonen et al. 2013). Also, forest owners should be seen as active agents rather than mere intermediaries who need to be trained (Matilainen et al. 2023). Empowering forest owners to retain decision-making power for themselves and their future generations would likely aid in reconciling the biodiversity and water protection targets with other objectives (Koskela and Karppinen 2024). Overall, the cooperation and dialogue among environmental advisors and specialists, forest advisors, and forest owners should be fostered (see also Vainio and Paloniemi 2012; Korhonen et al. 2013; Häyrinen et al. 2015; Mancheva 2018).

Applying a variety of means in the dissemination of information is needed to facilitate the decision-making process for forest owners (e.g. Toivonen et al. 2005; Hujala et al. 2007; Bergseng and Vatn 2009; Korhonen et al. 2013; Kuhlman et al. 2022; Takala et al. 2022; see also a review by Tiebel et al. 2022). If the aim is to target stream protection guidance cost-efficiently, actual protection efforts should be targeted to non-resident forest owners, who are more willing to consider biodiversity compared to resident owners. They are also more willing to accept advice from professionals (Hujala et al. 2009; Karppinen 2012; Karppinen and Berghäll 2015). In turn, our results suggest that resident owners would benefit from receiving information on combining forest management and biodiversity aspects. However, from an ecological perspective, protection should be targeted to sites with high natural values, and overall water protection demands effective water protection measures for all channels and water bodies. Thus, a minimum degree of mandatory regulation is probably necessary to meet the goals of enhancing biodiversity and water quality (see also Hysing and Olsson 2005; Danley et al. 2021).

As any survey, ours has potential weaknesses. Firstly, the response rate was lower compared to many other studies (e.g. Koskela and Karppinen 2024). Our response rate may not be of great concern, however, as the response rate does not necessarily bias the survey outcome (Hendra and Hill 2018)–more important is to determine whether the parameters of the group of respondents correspond well with the target population. The parameters we were able to compare (age, gender, and the size of owned forest estate) showed that the respondents correspond well with the population of the study area, except for the fact that there were fewer women respondents than would be expected from the population–a common phenomenon in surveys (e.g. Favada et al. 2009; Kuhlman et al. 2022). However, conducting a survey online may have reduced response rates compared to mail surveys (Shih and Fan 2009) and excluded respondents who, e.g. lack skills in using IT technology. This may have increased the proportion of positive answers to question related to information seeking activity. Also, e-mail addresses have probably been provided especially by forest owners who have participated in the training programs of Finnish Forest Centre. Thus, they may be on average more familiar with biodiversity and water protection subjects as some of the training programs focus on them. If so, our results would be biased towards positive attitudes towards safeguarding nature and may represent the future forest owner generation better than the present one (Juutinen et al. 2020; Husa and Kosenius 2021).

Overall, our results can be assumed to describe sufficiently the opinions of forest owners in North Ostrobothnia. The forest ownership structure, the state of headwater streams, and forestry intensity differ somewhat from northern compared to southern Finland as well as among Finland and other boreal countries, so our results may not directly mirror the situation in these other regions. Also, the differing legal status of streams and differences, e.g. in forest management history and the way forests are discussed in public, in other countries may restrict the generality of our results. However, the results provide indicative information about the situation in at least Finland and Sweden, which both rely heavily on voluntary commitments (including forest certification) on stream protection, and which have similar proportions of forested land, similar forest owner structures, and comparable forest use histories (Siiskonen 2013; Ring et al. 2017). They may also inform the attitudes of Canadian forest owners as forested buffers are not required along small streams (Kuglerová et al. 2020), yet Canada has considerably less privately owned forest land than Finland and Sweden. Further, there are similarities in the overall status of streams among the countries. Streams have undergone channelization and are therefore mostly ditch-like in Finland, Sweden, and most streams in these countries and Canada have degraded due to riparian loggings and cleaning the channels of boulders and large woody debris (Slaney and Martin 1997; Nilsson et al. 2014). This may affect how forest owners perceive streams in different regions: it can be expected that forest owners who have experienced nature beauty–that is, natural-like streams–are more engaged in safeguarding them (Diessner and Niemiec 2023).

Future research should aim to clarify the most effective way to increase the knowledge of forest owners about stream protection, considering factors such as age, gender, and place of residence. Also, the role of forest certification schemes deserves further attention as certificates have a significant impact on how riparian forests are managed. Either certificates should be developed to ensure that the protection of streams is not compromised, or forest owners who rely on certificates should be given further guidance on how to reduce the impact of forest management on streams. Further research is needed on the amount that forest owners are willing to invest in water protection in terms of lost revenue, and which kind of financial incentives would be necessary.

## Supplementary Information

Below is the link to the electronic supplementary material.

Supplementary file1 (PDF 449 KB)

## Data Availability

The raw questionnaire data are protected and are not available due to data privacy laws. The processed anonymized data set is available from authors upon reasonable request.
