# Supplementary material for: Family forest owner’s perspectives on headwater streams in boreal forests: Motivations, values, and conservation actions
Source: Ambio. 2025 Apr 5;54(9):1514–29. doi: 10.1007/s13280-025-02163-3 (PMC12307270; doi:10.1007/s13280-025-02163-3)
Supplement: Supplementary file 1 — Supplementary file1 (PDF 449 KB) [file 13280_2025_2163_MOESM1_ESM.pdf]

Ambio
Supplementary Information
This supplementary information has not been peer reviewed.

Title: **Family forest owners' perspectives on headwater streams in boreal forests: Motivations, values, and conservation actions**

Appendix S1. Explanations for classes 1-5 of stream naturalness

In the modelling data (Finnish Environment Institute 2021) streams were categorized in five classes:

1 = protection value low. The stream is strongly modified, and needs heavy channel and catchment restoration measures in order to be brought to higher class. The channel is ”ditch-like”, straightened and cleaned from boulders, stones and dead wood. The depth and width variation of the channel is extremely low or non-existent, and vegetation, shading and/or shelters are scarce. Typically there is heavy impairement due to land-use in the cathment.

2 = the state is heavily impaired. Usually can be restored with quite heavy channel and catchment restoration measures to class 3 or 4. The channel is ”ditch-like”, straightened and cleaned from boulders, stones and dead wood. The depth and width variation of the channel is low, and vegetation, shading and/or shelters are scarce. Typically there is heavy impairement due to land-use in the cathment.

3 = the state is impaired. Can be restored with channel and catchment restoration measures of different degrees to class 4. The channel lacks natural-like depht and width variation, and there is scarcity in vegetation, shading and/or shelters. Usually there is impairement due to land-use in the cathment.

4 = the state is only moderately impaired. Can be restored to class 5 with quite light channel and catchment restoration measures, or returns to its natural state over time. The channel has only slight impairment that is usually due to point-like modification on the channel and/or catchment.

5 = completely natural state. No impairement can be detected due to modification of the channel or the catchment.

Appendix S2. Forest owner survey

Mandatory questions marked with an asterisk (*)

Dear forest owner,

Water protection and consideration of forest biodiversity are part of forest management. The project "GIS-based planning of protection of riparian forests of headwater streams" aims to better identify the specific characteristics of the protection zones of streams and to develop the delimitation of the protection zones using spatial data. The aim is to promote favourable trends in both water quality and biodiversity.

A survey will be carried out to gauge the views of forest owners on the management of protection zones in commercial forests (excluding forest law sites) along streams. We welcome responses to the survey regardless of whether there is a stream on the forest plot you own or not. All responses are important. The participation of as many respondents as possible will ensure that the survey results provide a comprehensive and accurate picture of the opinions of forest owners in North Ostrobothnia. The project is a joint research project between Finnish Environment Institute, Natural Resources Institute Finland, Finnish Forest Centre and University of Oulu, funded through EU's Northern Finland Regional Development Fund. You can find more information about the project on our website.

In the survey

- A stream is a naturally formed watercourse smaller than a river. Streams may have been modified by human activity, for example straightened or dammed. In contrast, ditches originally dug by man are not streams.
- Stream protection zone means the riparian strip of a stream where forest management is different from the surrounding area or where no management is applied.

The participants in the survey have been selected from the Finnish Forest Centre's forest information system to include natural persons and estates owning more than 10 ha of forest land in

North Ostrobothnia. The data are processed in such a way that no individual respondent can be identified from the answers. Responding is voluntary. All the information you provide is confidential and will only be used for the purposes of the survey described above. **The information you provide will be treated anonymously** so that the identity of the respondents will not be revealed. By completing this form, you agree to the processing of your personal data in accordance with the Privacy Notice and you authorise the use of your data for research purposes within the Finnish Environment Institute.

Please complete the questionnaire below by 1 May 2022. If you wish, you can participate in a prize draw at the end of the survey (1 Fairphone 4 - 5G smartphone, worth approximately €600). **Participation in the draw** is voluntary.

For more information on the survey, please contact Mari Annala (mari.annala@syke.fi) and Virpi Lehtoranta (virpi.lehtoranta@syke.fi).

Heikki Mykrä, project manager, Finnish Environment Institute SYKE

1. In which municipalities are Your forest holding(s) currently located? You can select more than one option if necessary.

Alavieska, Haapajärvi, Haapavesi, Hailuoto, Ii, Kalajoki, Kempele, Kuusamo, Kärsämäki, Liminka, Lumijoki, Merijärvi, Muhos, Nivala, Oulainen, Oulu, Pudasjärvi, Pyhäjoki, Pyhäjärvi, Pyhäntä, Raahe, Reisjärvi, Sievi, Siikajoki, Siikalatva, Taivalkoski, Tyrnävä, Utajärvi, Vaala, Ylivieska

2. Total forest area owned by You (ha). Please answer with an even number, without decimal point (e.g., 50). An estimate is sufficient if you do not have exact data.

3. What is the main type of ownership of Your forest holdings?

I own the holding alone / I own the holding jointly with one or more persons / the holding is a group / the holding is an inheritance / other, which?

4. Please select the option that best describes Your situation

I live on the forest holding / I live in the same municipality as where my forest holding(s) are located / I live in a different municipality from where my forest holding(s) are located

5. Have You made a timber trade in the last 3 years?

yes / no / don't know

6. How much do the following forest-related issues influence Your desire to own a forest?

Please tick one box for each statement

Not at all / Not very much / Neutral / Quite a lot / Very much / Don't know

a. Source of main income or regular additional income
b. Financial security in case of unexpected expenses
c. Investment
d. A legacy for children
e. Plot of land for a permanent or holiday home
f. Gain of firewood
g. Exercise in forestry activities
h. Recreational use of forests (berry picking, hunting, nature walks and outdoor recreation).
i. Landscape
j. Conservation of biodiversity (e.g., leaving decaying trees in the forest)
k. Nature conservation (e.g., protection through a nature reserve or in METSO-programme)
l. Production of special natural products (e.g., sap, spruce sprouts)
m. Carbon sequestration

7. How many <u>hectares</u> of Your forest holding(s) have been protected (e.g., nature reserve, METSO or other specified protection)? Please answer with numbers (e.g., 50). An estimate is sufficient if there´s no exact data.

8. Is any of Your forest holding(s) included in the forest certification systems? Please select only one option.

a. My forest is certified under the PEFC certification scheme
b. My forest is certified under the FSC certification scheme
c. My forest is certified under both the PEFC and the FSC certification schemes
d. My forest is not included in the certification scheme
e. I do not know if my forest is certified

9. Do any of Your forest holdings have the following types of forests bordering waterbodies? You can select more than one option if necessary. Here, streamside forest refers to the forest area that reaches from the edge of the stream channel to 30 meters from the stream.

Yes / No / Don't know

a. Lake or pond riparian forest
b. Sea shore
c. Riverside forest (average width of the channel is at least 5 m)
d. Streamside forest (with an average width of the channel 5 m or less) *

—► Please consider the following questions for Your forest holdings of more than 10 ha in North Ostrobothnia.

10. Do(es) Your forest holding(s) have forest law sites along a stream? Here, streamside forest refers to the forest area that reaches from the edge of the stream channel to 30 meters from the stream. A forest law site is a forest area of biodiversity value according to §10 of the Forest Act.

no / yes / don't know

11. What kind of protection zones have been left in Your streamside forests in case of possible loggings during your ownership? You can select more than one option if necessary. Here, streamside forest refers to the forest area that reaches from the edge of the stream channel to 30 meters from the stream. Stream protection zone refers to the riparian strip of the stream where forest treatment differs from the surrounding area, or no treatment is applied.

a. No logging has taken place on the riparian area
b. A 5-10 m equal width buffer has been retained along the stream(s)
c. A 15-metre equal width buffer has been retained along the stream(s)
d. A buffer zone of more than 15 metres has been retained along the stream(s)
e. A buffer zone of varying width has been retained along the stream(s)
f. No buffer zones have been retained along the stream(s)
g. Other, what kind?
h. I do not know what kind of protection zones have been left along the stream(s)

12. How have the forest been treated on the possible loggings during Your ownership? You can select more than one option if necessary. Here, streamside forest refers to the forest area that reaches from the edge of the stream channel to 30 meters from the stream. Riparian buffer means the riparian area of the stream where forest treatment is different from the surrounding area, or no treatment is applied.

a. No logging has taken place along the streams
b. All the trees of the riparian buffer have been retained in the loggings
c. The forest in the stream protection zone has been thinned
d. Deciduous trees, undergrowth and shrub layer have been retained in the riparian buffer
e. Only retention trees and shrub layer have been retained in the riparian buffer

f. No trees have been retained in the riparian buffer, but tree seedlings and shrub layer have been retained
g. In other ways, how?

13. If Your streamside is to be logged, what kind of buffer zone would You prefer? Please choose the most likely option. Here, streamside forest refers to the forest area that reaches from the edge of the stream channel to 30 meters from the stream. Riparian buffer means the riparian area of the stream where forest treatment is different from the surrounding area, or no treatment is applied.

a. No logging along the stream is planned
b. Variable-width, 10 m on average (minimum of 5 m)
c. Variable-width, width could vary from 5 m to 30 m
d. Fixed-width of 10 m
e. Fixed-width of 15 m
f. Fixed-width of 30 m
g. Other, what kind?

14. If Your streamside is to be logged, how would you like the forest in the protection zone to be treated? You can select more than one option if necessary. Here, streamside forest refers to the forest area that reaches from the edge of the stream channel to 30 meters from the stream. Riparian buffer zone means the riparian area of the stream where forest treatment is different from the surrounding area, or no treatment is applied.

a. No logging along the stream is planned
b. All trees will be retained in the riparian buffer zone
c. Thinning of the forest in the riparian buffer zone is possible
d. The forest in the riparian buffer zone may be selectively harvested, with the aim of natural regeneration of the stand
e. Concentration of retention trees groups in the riparian buffer zone

f. All deciduous trees, undergrowth and shrub layer shall be retained in the riparian buffer zone
g. All trees may be removed from the riparian buffer zone, except for the retention trees
h. The proportion of deciduous trees in the riparian buffer zone shall be increased
i. In other ways, how

15. How do the following statements describe Your activities in the near future (1-5 years) on Your forest holdings of more than 10 ha in North Ostrobothnia? In continuous cover forestry, there are always trees in the forest and no clear-cutting is done.

Totally disagree / Somewhat disagree / Neither agree nor disagree / Somewhat agree / Totally agree / I don't know

a. I intend to use continuous cover forestry on appropriate sites.
b. I intend to use even-aged forestry
c. I intend to actively seek information on different forest management options
d. I intend to actively seek information on available information and/or latest recommendations on forest biodiversity enhancement
e. I intend to take better into account the water protection objectives
f. I intend to compare different forest management options before deciding on the forest management
g. I intend to choose an option recommended by a forest plan or a forestry professional
h. I will not be able to make decisions about forest management activities without further information
i. I need more information on forest certification criteria for stream riparian site treatment
j. I need more information on the measures recommended in the forest management recommendations before deciding on forest treatment

16. If You wish, You may justify your choices in question 15:

17. How do You agree or disagree with the following statements? Please consider the whole of the North Ostrobothnia region. A stream is a naturally formed watercourse smaller than a river. Streams may have been modified by human activity, for example straightened or dammed. In contrast, ditches originally dug by man are not streams. Diversity here refers to the abundance of species, the genetic variability of species and the diversity of habitats.

Strongly disagree / Somewhat disagree / Neither agree nor disagree / Somewhat agree / Strongly agree / Don't know

a. It is important that water quality of streams does not deteriorate
b. It is important that streams have a diverse biota
c. It is important that streamside forests are thriving
d. It is important to protect endangered brown trouts
e. The scenic value of the stream is important
f. Streams are important for human well-being (e.g., as recreational areas)
g. In my opinion the state of the streams is good enough at the present
h. Forestry activities in the immediate vicinity of the stream compromise the water quality
i. The biodiversity of the streams will be compromised if the streamside forests are logged
j. Forest owners must also take responsibility for the condition of watercourses on their forest holdings
k. The forest owner is not responsible for the development of the biodiversity of streams and riparian areas
l. Monetary compensation must be given to the forest owner for the work done for the biodiversity of streams and riparian areas

Background information

18. Year of birth (e.g., 1966) *

19. Your gender *

male / female / other / do not want to say

20. Main place of residence during Your childhood *

rural / urban or urban environment / municipal centre

21. Your highest level of education *

primary school degree / secondary school degree / high school diploma / bachelor's degree / master's degree / doctoral degree / other, which?

22. Are You or have You been gainfully employed in the forestry or agricultural sector? *

yes /no

23. Free to speak! Anything left unsaid or any questions that came to mind?

24. Would You like to enter a prize draw to win a Fairphone 4 - 5G smartphone (€589)? To enter, please provide Your contact details. The personal data will only be used to run the draw and to contact the winner of the draw. *

Yes / No
